# NMDA Mediated Contextual Conditioning Changes miRNA Expression

**DOI:** 10.1371/journal.pone.0024682

**Published:** 2011-09-12

**Authors:** Min Jeong Kye, Pierre Neveu, Yong-Seok Lee, Miou Zhou, Judith A. Steen, Mustafa Sahin, Kenneth S. Kosik, Alcino J. Silva

**Affiliations:** 1 Neuroscience Research Institute, University of California Santa Barbara, Santa Barbara, California, United States of America; 2 Department of Molecular, Cellular and Developmental Biology, University of California Santa Barbara, Santa Barbara, California, United States of America; 3 Kavli Institute for Theoretical Physics, University of California Santa Barbara, Santa Barbara, California, United States of America; 4 Departments of Neurobiology, Psychiatry and Psychology, Brain Research Institute, Integrative Center for Learning and Memory, David Geffen School of Medicine at University of California Los Angeles, Los Angeles, California, United States of America; 5 The F.M. Kirby Neurobiology Center, Department of Neurology, Children's Hospital Boston, Harvard Medical School, Boston, Massachusetts, United States of America; Emory University, United States of America

## Abstract

We measured the expression of 187 miRNAs using quantitative real time PCR in the hippocampal CA1 region of contextually conditioned mice and cultured embryonic rat hippocampal neurons after neuronal stimulation with either NMDA or bicuculline. Many of the changes in miRNA expression after these three types of stimulation were similar. Surprisingly, the expression level of half of the 187 measured miRNAs was changed in response to contextual conditioning in an NMDA receptor-dependent manner. Genes that control miRNA biogenesis and components of the RISC also exhibited activity induced expression changes and are likely to contribute to the widespread changes in the miRNA profile. The widespread changes in miRNA expression are consistent with the finding that genes up-regulated by contextual conditioning have longer 3′ UTRs and more predicted binding sites for miRNAs. Among the miRNAs that changed their expression after contextual conditioning, several inhibit inhibitors of the mTOR pathway. These findings point to a role for miRNAs in learning and memory that includes mTOR-dependent modulation of protein synthesis.

## Introduction

Ever since new protein synthesis was proposed as a requirement for memory formation [1], many different molecular mechanisms related to transcription, translation and post-translational modifications have been implicated in learning and memory [2]. Synaptic plasticity and memory storage require precise regulation of gene expression and spatiotemporally constrained protein synthesis near synaptic sites [3].

MicroRNAs (miRNAs) are small non-coding RNA molecules that can dampen the expression of specific proteins by binding to the 3′-untranslated regions (UTR) of their target genes [4], [5]. These miRNA-mRNA duplexes are housed within the RNA-induced silencing complex (RISC). Binding of the RISC to mRNAs blocks translation or destabilizes mRNAs [6]. Each miRNA can regulate protein levels, often by small amounts, encoded by hundreds of genes directly or indirectly [7], [8]. miRNAs regulate a broad range of cellular functions, such as stem cell maintenance [9], cellular differentiation [10], [11], synaptic plasticity [12], [13] and learning and memory processes [14].

The mechanism of miRNA regulation at the synapse involves activity-dependent degradation of a protein in the RISC known as Armitage in Drosophila [15] or Mov10 in mammals [16]. In these examples, the silenced mRNA is de-repressed and translated due to the loss of RISC integrity. Many miRNAs are present in neurites of neurons suggesting that miRNAs might play an important role in the control of local translation at synaptic sites [17], [18]. An increasing number of reports describe individual miRNAs that regulate translation of genes at synapses. For example, miR-134 regulates LimK1 at the spine by stimulation of BDNF [19], miR-138 regulates palmitoylation in neurons by inhibiting the translation of LYPLA [16], [18], miR-132 targets p250GAP to enhance spine growth [20] and the FMRP associated miRNA, miR-125b blocks the translation of NR2B resulting in neuronal structural changes [21]. In *Aplysia californica*, miR-124 was rapidly down regulated on stimulus of serotonin constrained synaptic facilitation through regulation of transcription factor, CREB [22]. In mouse, the deacetylase SIRT1 modulates learning and synaptic plasticity via miR-134, which represses translation of BDNF and CREB [14], [19]. Conditional disruption of Dicer1, which processes pre-miRNAs to mature miRNAs, enhanced learning and memory and changed neuronal morphology [23]. Transient down regulation of Ago2 protein in the dorsal hippocampus impaired contextual conditioning [24]. Haploinsufficiency of DGCR8, a component of the microprocessor complex, caused altered short-term plasticity and cognitive deficits in a mouse model [25], [26]. Although those studies suggest that miRNAs have a role in learning and memory formation, a systems approach to miRNA regulation in learning and memory has not been reported.

Here, we report miRNA expression profiles in the hippocampal CA1 region after contextual conditioning as well as expression changes in genes, which are involved in miRNA biogenesis. We suggest that miRNAs and the genes associated with their biogenesis play a role in learning and memory in the rodent hippocampus.

## Results

### Contextual conditioning triggers NMDA-dependent changes in miRNA expression in mice

**Figure 1:**
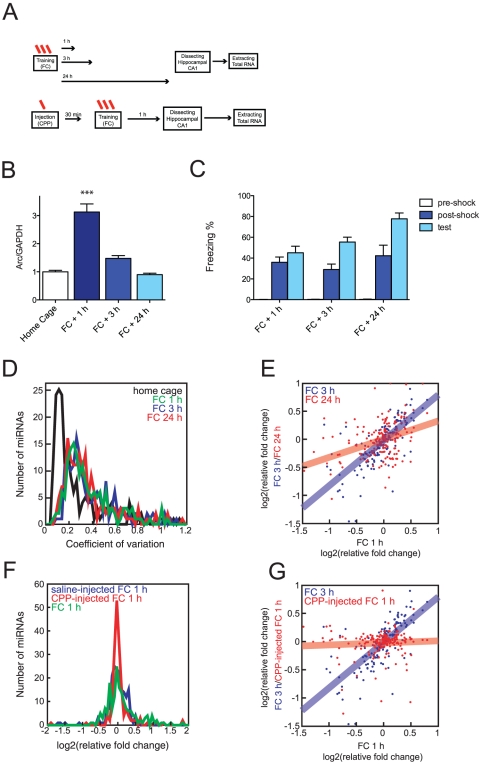
Contextual conditioning induces NMDA-dependent wide scale changes in the global miRNA profile. (A) Schematic of the experimental design. Mice were trained and memory tested. CA1 region was removed at 1, 3 and 24 hours after fear conditioning (FC). CPP was injected 30 min prior contextual conditioning. (B) Relative Arc transcript levels in CA1 region after training (*** p<0.001, t-test, home cage: n = 12, 1 h and 3 h: n = 12, 24 h: n = 8, mean ± S.E.M.) (C) Contextual fear memory tests at 1, 3, and 24 hr after training. Freezing before (pre-shock) and immediately after shock (post-shock) during the training are also shown. (D) Coefficient of variation of 184 miRNA levels from CA1 region of home caged or fear conditioned animals (1, 3 and 24 hours after training). miRNA levels were more heterogeneous after FC (p<10^−14^, Kruskal-Wallis test). (E) Comparison of the relative expression changes for individual miRNAs at 3 (blue dots and blue line) and 24 h (red dots and red line) after FC with 1 h after FC. Solid lines are linear fits of the data. Changes were correlated between 1 and 3 h (rS = 0.71, p = 3.10^−26^) and 24 h (rS = 0.38, p = 7.10^−7^). (F) Relative mean expression changes of 187 miRNAs 1 h after FC with an injection of a saline solution (blue), of CPP, an NMDA receptor antagonist (red) or without any injection (green). Levels were compared to those of naive animals. Saline-injected and CPP-injected distributions were significantly different (p = 0.02, Kolmogorov-Smirnov test), the distribution of CPP-injected profile is sharper than the saline-injected one (p<10^−6^, F-test). (G) Comparison of the expression changes 1 h after FC with 3 h after FC (blue dots and blue line) or with injection of the NMDA receptor antagonist, D(–)-3-(2-carboxypiperazine-4-yl)-propyl-1-phosphonic acid (CPP) (red dots and red line). Levels were compared to those of naive animals. Changes in the presence of CPP were minimal and uncorrelated with the ones in conditioned animals (r = 0.06, p = 0.44). The solid line is a linear fit of the data.

To study the miRNAs that undergo expression changes with learning, we used contextual conditioning. Mice were trained with three foot-shocks in a training chamber. Animals were sacrificed at several time points after training (1, 3 and 24 h), and the hippocampal CA1 region was dissected (Figure 1A). Before the mice were euthanized, we tested contextual conditioning to ensure that they acquired the contextual conditioning task (Figure 1C). In addition, the expression of Arc was used as a positive control (Figure 1B). [27], [28]

We assayed the expression levels of the mature form of 187 miRNAs in the hippocampal CA1 region of individual animals using quantitative real time PCR (Data S1). Among 20 naive mice, animal to animal variability in the expression of 184 detected miRNAs was surprisingly small. The median coefficient of variation was 0.17. (Figure 1D, Black line) This indicates that miRNA expression in the adult mouse hippocampal CA1 region is very homogenous, and that our method to measure miRNA expression is reliable. We also confirmed that the assessment of the freezing behavior before sacrificing animals did not affect miRNA expression (data not shown). After contextual conditioning, the variability in the expression of the majority of miRNAs studied increased significantly. At each of three different time points, the median coefficient of variation was 0.32–0.36 (p<10^−14^, Kruskal-Wallis test, Figure 1D). This indicates that contextual conditioning changes miRNA expression, and the effect lasts at least 24 hours.

We compared the expression of individual miRNAs between naive (home cage, HC) and three different time points (1, 3 and 24 h) after contextual conditioning (FC) (Figure 1E). The X-axis of Figure 1E plots the difference between 1 h after contextual conditioning (1 h) and HC, and the Y-axis plots the difference between 3 h after contextual conditioning (3 h) and HC (blue) and the difference between 24 h after contextual conditioning (24 h) and HC (red). Both (3 h and 24 h) are correlated with 1 h, but correlation between 1 h and 3 h is stronger than 1 and 24 h. (between 1 and 3 h; r_S_ = 0.71, p = 3.10^−26^, between 1 and 24 h; r_S_ = 0.38, p = 7.10^−7^). In other words, a defined set of miRNAs changed after contextual conditioning, and these changes are most similar between 1 and 3 hours. These data indicate that the miRNA expression changes after contextual conditioning are not random. Furthermore, the differences in miRNA expression between short term responses (1 h and 3 h) and long-term responses (24 h) raise the possibility that there are two different miRNA-mediated mechanisms underlying contextual conditioning [29].

To address the mechanism by which miRNA expression is regulated following contextual conditioning, we determined the impact of blocking NMDA-receptors with intraperitoneal injections of D(–)-3-(2-carboxypiperazine-4-yl)-propyl-1-phosphonic acid (CPP) 30 minutes before training (Figure 1A). Blocking NMDA receptors is known to result in impairments in certain forms of hippocampal plasticity and in profound contextual conditioning deficits [30]. We confirmed that contextual conditioning was successfully blocked by CPP injections (data not shown). The expression of miRNAs was measured 1 h after contextual conditioning. CPP injected animals showed smaller changes in miRNA expression compared to saline injected animals after contextual conditioning (Figure 1F). The miRNA expression of CPP-injected, fear- conditioned animals also differed from fear- conditioned animals without injection (Figure 1F). miRNA expression at 1 h after contextual conditioning showed very low correlation with CPP injected contextually conditioned animals (Figure 1G). This finding indicates that the majority of changes we found in the expression of miRNAs after contextual conditioning are NMDA receptor dependent and are likely related to learning in the contextual conditioning task.

**Figure 2:**
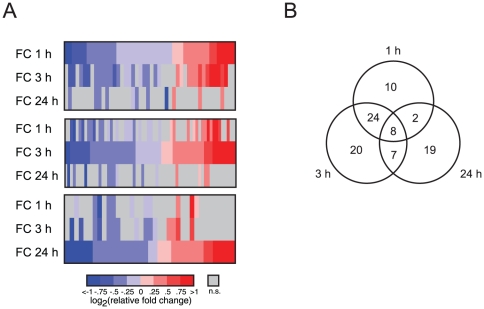
miRNA temporal expression patterns after conditioning in hippocampal CA1 region. (A) Three heatmaps of miRNA expression after contextual conditioning. They are organized based on time after contextual conditioning. up; 1 h, middle; 3 hrs, down; 24 hrs. (colored miRNAs are statistically significant, n.s. =  not significant). (B) Diagram of miRNAs changing after contextual fear conditioning (FC) (p<0.05, Kruskal-Wallis test, home cage: n = 20, 1 h and 3 h: n = 12, 24 h: n = 8.)

Analyses of expression trends across the entire population of miRNAs suggested two possible patterns of miRNA expression. The median expression of 44, 59 and 36 miRNAs was significantly changed at 1, 3 and 24 h after contextual conditioning, respectively (Kruskal-Wallis test, p<0.05, Figure 2A and B, Figure S1 and Table 1). Among the miRNAs that undergo the greatest dynamic regulation at early time points were miR-219 and miR-140 (Figure S1A) and at the later time point were miR-322 and miR-128a (Figure S2B, 3 and 24 h). Some miRNAs such as miR-24, miR-186, let-7f and miR-320 showed changes in expression throughout all time points (Figure S2C). All the miRNAs in this category showed the same direction of change.

**Figure 3:**
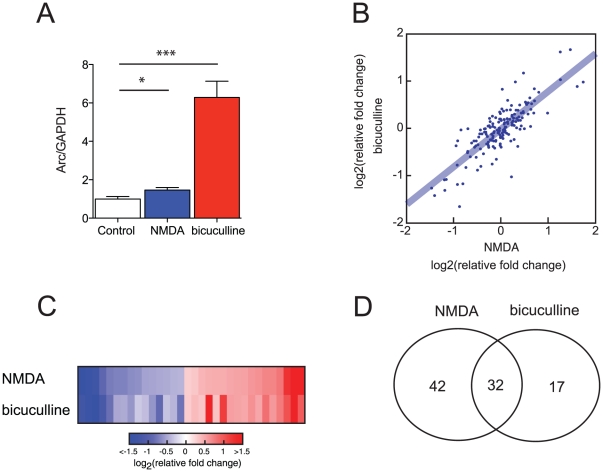
miRNA expression patterns 1 hour after chemical stimulation with NMDA and bicuculline to 15 days cultured rat embryonic hippocampal neurons. (A) Relative ARC transcript levels are increased after stimulation with NMDA or bicuculline. (*p<0.05, ***p<0.001, t-test, mean ± S.E.M.). (B) Mean relative fold changes compared to unstimulated neurons were computed for each stimulation method. The amplitude of expression changes was remarkably correlated. (rS = 0.81, p = 7.10^−41^). (C) miRNAs with expression significantly different from the one in unstimulated neurons. (p<0.05, Kruskal-Wallis test). (D) 32 miRNAs showed expression changes in common after NMDA and bicuculline stimulation. (unstimulated; n = 10, NMDA; n = 10, 60 µM, 5 min treatment, bicuculline; n = 8, 20 µM, 1 hour treatment).

**Table 1:** List of miRs changing after fear conditioning.

Time after contextual conditioning	miRs
1 hr	let-7d, miR-139, miR-145, miR-146, miR-15b, miR-16, miR-181a, miR-186, miR-196b, miR-19a, miR-21, mir-214, miR-219, mir-223, miR-24, miR-27a, miR-29b, miR-30b, miR-30c, miR-30e, miR-320, miR-342, miR-92, let-7f, miR-140, miR-99a, miR-10b, miR-129*, miR-140*, miR-151, miR-211, miR-217, miR-291-3p, miR-298, miR-300, miR-329, miR-336, miR-337, miR-345, miR-346, miR-347, miR-429, miR-483, miR-7*
3 hrs	let-7d, miR-107, miR-10a, miR-126, miR-128a, miR-135b, miR-139, miR-145, miR-146, miR-148b, miR-152, miR-15b, miR-16, miR-17, miR-184, miR-185, miR-186, miR-193, miR-19a, miR-19b, miR-200b, miR-21, miR-213, miR-219, miR-24, miR-27a, miR-30b, miR-30c, miR-320, miR-34c, miR-433, let-7f, miR-134, miR-140, miR-203, miR-204, miR-99a, miR-10b, miR-129*, miR-140*, miR-151*, miR-211, miR-291-3p, miR-298, miR-300, miR-301, miR-322, miR-325, miR-326, miR-329, miR-341, miR-345, miR-346, miR-347, miR-351, miR-429, miR-483, miR-7*, miR-93
24 hrs	let-7e, miR-100, miR-106b, miR-125b, miR-128a, miR-128b, miR-130a, miR-142-5p, miR-17, miR-186, miR-22, miR-222, miR-24, miR-25, miR-26a, miR-29a, miR-30e, miR-320, miR-338, mir-339, miR-34a, mir-449, let-7f, miR-134, miR-203, miR-217, miR-31, miR-322, miR-326, miR-345, miR-346, miR-424, miR-483, miR-7, miR-7*, miR-93

miR-134 was one of the miRNAs that changed at the late time point. This miRNA is thought to regulate neuronal morphology by targeting LimK1 and its expression is regulated by BDNF in neurites [19]. Recently, it was also shown that miR-134 regulates learning and memory in a mouse model [14]. We have also noticed that miRNAs previously detected in neurites such as miR-134, miR-25 and miR-26a showed changes at 24 h after contextual conditioning [17], [19].

### Stimulation in cultured hippocampal neurons using NMDA and bicuculline changes miRNA expression

We stimulated DIV15 cultured embryonic rat hippocampal neurons with NMDA (60 µM, 5 min) or bicuculline, an antagonist of GABA receptors (20 µM, 1 h). One hour after stimulation, we collected samples and measured miRNA expression (Data S2). Expression of Arc was measured in the same RNA samples as a positive control (Figure 3A). The amplitude of expression changes, i.e., the set of Δ Ct's, was remarkably correlated in the two different stimulation methods (r_S_ = 0.81, p = 7.10^−41^) (Figure 3B). Most of the miRNAs that changed were up- or down-regulated by small amounts; however, a few changed by as much as 3-fold after stimulation. The strong correlation in the amplitude of the miRNA response after two different experimental conditions, NMDA and bicuculline, suggests that neuronal stimulation elicits a global coordinated change in miRNA expression. 32 miRNAs showed significant changes in their median expression levels in both NMDA and bicuculline treated neurons (Figure 3C, D, Figure S2 and Table 2). Among the differentially expressed miRNAs was miR-132, a known activity-dependent miRNA [20].

**Table 2:** List of miRs changing after neuronal stimulation.

Stimulation methods	miRs
NMDA	miR-347, miR-340, miR-205, miR-107, miR-217, miR-327, miR-483, miR-129*, miR-333, miR-187, miR-320, miR-132, miR-292-5p, miR-10b, miR-335, miR-451, miR-140*, miR-344, miR-326, miR-291-3p, miR-193, miR-292-3p, let-7e, miR-301, miR-337, let-7c, miR-29b, miR-290, miR-212, miR-204, let-7a, let-7d, miR-191, miR-433, miR-16, miR-351, miR-181c, miR-22, miR-151*, miR-25, miR-106b, miR-27b, miR-424, miR-31, miR-19b, miR-324-3p, miR-28, miR-148b, miR-18, miR-213, miR-126, miR-99b, miR-450, miR-23a, miR-24, miR-203, miR-221, miR-328, miR-30e, miR-21, miR-30a-3p, miR-329, miR-200a, miR-139, miR-338, miR-152, miR-151, miR-27a, miR-298, miR-190, miR-34a, miR-146, miR-138, miR-153
bicuculline	miR-205, miR-219, miR-340, miR-218, miR-347, miR-483, miR-183, miR-193, miR-187, miR-29b, miR-96, miR-126*, miR-326, miR-10b, miR-199a, miR-320, miR-132, let-7c, miR-92, miR-336, miR-344, miR-292-3p, miR-335, miR-181a, miR-350, miR-31, miR-99b, miR-365, miR-24, miR-324-3p, miR-140, miR-21, miR-338, miR-30e, miR-328, miR-27a, miR-329, miR-298, miR-33, miR-151, miR-153, miR-323, miR-7, miR-34a, miR-221, miR-142-3p, miR-146, miR-196b, miR-23a

### A set of up-regulated miRNAs common to conditioning and neuronal stimulation in culture target the mTOR pathway

**Figure 4:**
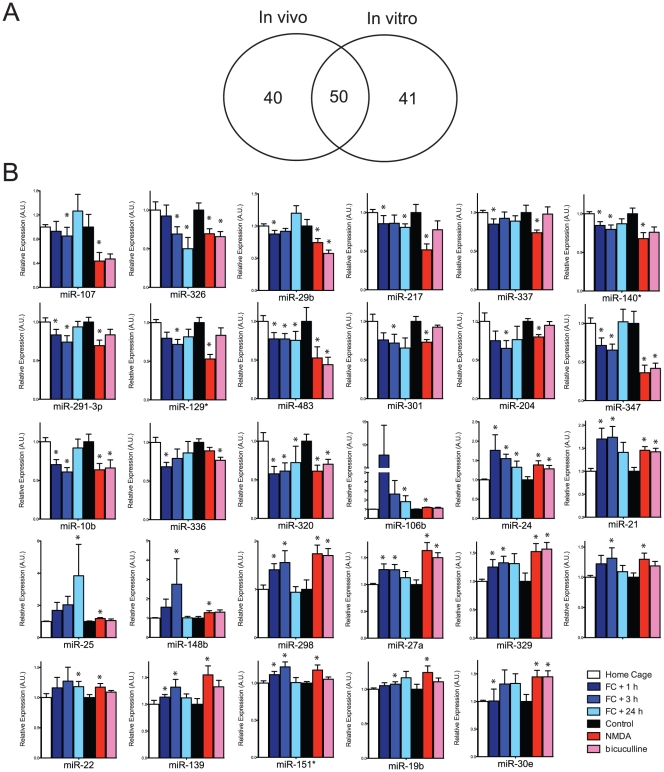
The expressions of 29 miRNAs are changed in vivo and in vitro in response to contextual conditioning and neuronal stimulation. (A) Diagram of miRNAs changing their expression after contextual fear conditioning (FC) and neuronal stimulation. 90 miRNAs are changing after FC *in vivo* in the CA1 region and 91 miRNAs are changing after neuronal stimulation in the culture system. (B) 14 miRs are increased and 15 miRs are decreased in both FC and neuronal stimulation at least in one condition per group. Statistical significance was marked with asterisk. (* p<0.05, Kruskal-Wallis test).

Among 187 miRNAs in our data set, 90 miRNAs were changed after contextual conditioning in at least one time point, and 91 miRNAs were changed after neuronal stimulation by either NMDA or bicuculline. (Figure 4A) Out of 50 miRNAs, which are common between the two sets of miRNAs, the expression of 29 miRNAs were changed in same direction (Figure 4B). Among these miRNAs with shared directions of change in *in vitro* cultured hippocampal neurons and *in vivo* hippocampal CA1 regions after either neuronal stimulation or contextual conditioning were miR-24, miR-326, miR-320, miR-21 and miR-10b. Interestingly, among 14 miRNAs, whose expressions are increased after contextual conditioning and neuronal stimulation, 7 of them were reported to increase mTOR dependent protein synthesis by targeting endogenous mTOR inhibitors (Table 3). For example, miR-106b, miR-21, miR- 22, miR-19b and miR-25 are known to regulate PTEN and miR-27 and miR-139 repress FoxO1 translation through direct binding to the 3′-UTR [31], [32], [33], [34], [35], [36], [37], [38]. miR-329 is also reported to regulate dendritic outgrowth in an activity dependent manner [39]. Among the up-regulated miRNAs, miR-106b, miR-25 and miR-19b share the same primary transcripts, and miR-24 and miR-27 share primary transcripts. The 15 down-regulated miRNAs didn’t appear to target a clear common pathway among validated target studies. (Figure 4B and Table 4)

**Table 3:** Upregulated miRs.

	Target genes	Pathways and References
miR-106b	TGF-b2, APP, p21, PTEN[35], [51], [52], [53]	Alzheimer's, ASD,
miR-24	E2F2, Myc [54]	
miR-21	PTEN, [33], [55]	Cell cycle, growth, mTOR
miR-25	FXR1P, MITF, PTEN [32], [35], [56]	mTOR
miR-148b	MITF, DNMT3b [57], [58], [59]	
miR-298	BACE [60]	Alzheimer's
miR-27b	Mef2c, PPARgamma, MMP13, Pax3, FoxO1 [38], [59], [61], [62], [63], [64]	mTOR
miR-329		Activity dependent dendrite outgrowth [39]
miR-213		
miR-22	PTEN/AKT, MYCBP [36], [65]	mTOR, myc pathway
miR-139	FoxO1 [31]	mTOR
miR-151*		
miR-19b	PTEN, [37]	mTOR
miR-30e		Schizophrenia [66]

**Table 4:** Downregulated miRs.

miRs		
miR-107	Granulin/progranulin, BACE [67], [68]	Neurodegenerative disease, Alzheimers', brain injury, Schizophrenia[69]
miR-326	Notch [70]	
miR-29b	DMNT3a,b, CDC42, [71], [72]	Rett syndrome (MeCP2), Alzheimers', fromtotemporal dimentia, [73], [74], [75]
miR-217	PTEN [76]	mTOR
miR-337		
miR-140*	DMN1 [77]	Synaptic endocytosis, nicotine addiction
miR-291-3p		
miR-129*		
miR-483		IGF [78]
miR-301	MEOX2 [79]	ERK/CREB
miR-204	Meis2, SIRT1 [80], [81]	Lens and Retinal development, embryonic stem cell differentiation
miR-347		
miR-10b	NF1, Tiam1 [82], [83]	Tumorigenesis, Carcinoma migration
miR-336		
miR-320	Transferrin receptor, CD71 [84]	IGF

### The expression of mature and pri-miRNAs can be uncoupled

**Figure 5:**
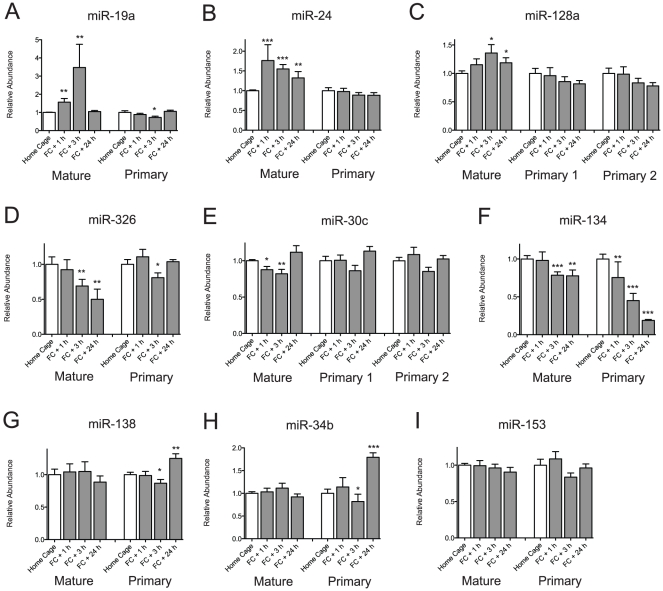
miRNA and pri-miRNA expression levels are largely uncoupled. (A–C) Up-regulated mature miRNAs have unchanged pri-miRNA levels. (D, E) Down-regulated mature miRNAs showing relatively unchanged pri-miRNA levels, (F) Both mature and pri-miRNA levels are down-regulated (G, H) Only pri-miRNAs are up-regulated (I) Both mature and pri-miRNAs are not changed after fear conditioning (FC). (* p<0.05, Man-Whitney test, home cage: n = 12, 1 h and 3 h: n = 12, 24 h: n = 8, mean ± S.E.M.).

Approximately 50% of the assayed miRNAs were changed after contextual conditioning. These changes could result from altered transcription of the miRNAs involved or altered downstream biogenesis and degradation pathways. We measured the expression of 11 primary transcripts and nine mature miRNAs from the same RNA samples of fear conditioned animals. In the case of miR-30c and miR-128a, the mature miRNAs map to two different primary transcripts. We measured both primary transcripts of these two miRNAs. Some up-regulated miRNAs such as miR-19a, miR-24 and miR-128a were unchanged at the level of their primary transcripts. (Figure 5A to C). Similarly, some down-regulated miRNAs, such as miR-326 and miR-30c, were also associated with no change in their primary transcripts (Figure 5D and E). On the other hand, miR-134, miR-138 and miR-34b showed bigger changes in the expression of their primary transcripts than their mature forms (Figure 5F to H). Especially, in the case of miR-134, about 20% of the mature form decreased in 24 hours after contextual conditioning, but 80% of primary transcript was decreased at the same time point. Mature forms of miR-138 and miR-324b were not changed after contextual conditioning at any time point, but their primary transcripts were significantly decreased at 3 hours and increased at 24 hours after contextual conditioning. miR-153 did not show any changes in either the expression of the primary transcript or the mature form (Figure 5I). These data show that the expression of the primary transcript and the mature form of the miRNA are not always coupled and the miRNA biogenesis pathway could be involved in contextual conditioning in the hippocampal CA1 region.

### Transcripts which control miRNA biogenesis and the RISC are regulated upon contextual conditioning

**Figure 6:**
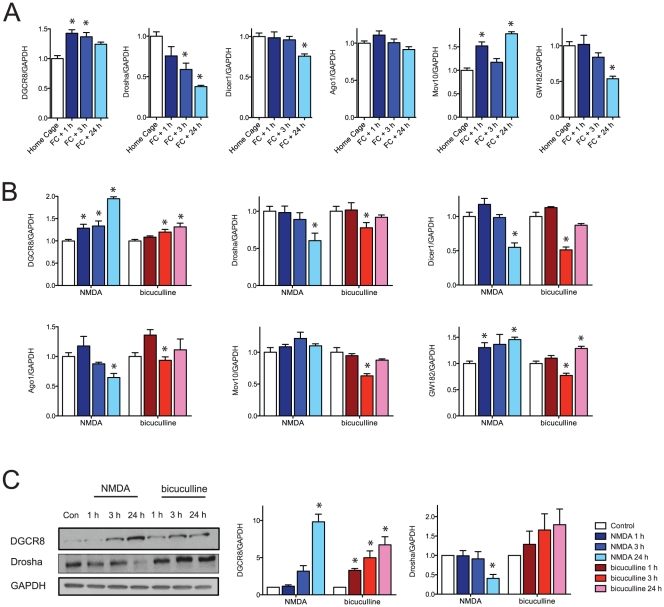
Contextual conditioning affects transcript levels of miRNA biogenesis enzymes and RISC components. (A) Transcript levels of DGCR8, Drosha, Dicer1, Ago1, Mov10 and GW182 *in vivo* hippocampal CA1 region after contextual fear conditioning. (*p<0.05, ANOVA, home cage: n = 12, 1 h and 3 h: n = 12, 24 h: n = 8. normalized by GAPDH, mean ± S.E.M.) FC =  fear conditioning. (B) Transcript levels of DGCR8, Drosha, Dicer1, Ago1, Mov10 and GW182 in *in vitro* cultured neurons after stimulation with NMDA or bicuculline. (*p<0.05, t-test, n = 8 per group from four different biological samples, normalized by GAPDH, mean ± S.E.M.) (C) Protein levels of DGCR8 and Drosha after neuronal activation. (*p<0.05, t-test, n = 3 from three different biological samples, normalized by GAPDH, mean ± S.E.M.).

We measured transcripts of genes in the miRNA pathway in the same RNA samples using the miRNA measurements. Transcripts encoding DGCR8, Drosha, Dicer, MOV10 and GW182 were measured in *in vivo* and *in vitro* samples. Interestingly, the miRNA biogenesis genes—DGCR8, Drosha, and Dicer—showed different patterns of expression after contextual conditioning. DGCR8 mRNA was increased at the early time point, Drosha mRNA gradually decreased and Dicer mRNA showed a small but significant decrease at the 24 h time point (Figure 6A). A similar trend was observed in cultured neurons. Changes in the protein levels of DGCR8 and Drosha following NMDA stimulation paralleled the changes in their transcript levels. However, stimulation with bicuculline showed different gene expression patterns (Figure 6B and C). Taken together these findings suggest a convergence in the miRNA regulatory pathways related to NMDA stimulation and contextual conditioning. The complex pattern of miRNA changes likely reflects the multiple mechanisms of miRNA biogenesis (and probably degradation) in the brain [40], which appear to include setting the ratio between DGCR8 and Drosha [41].

We measured expression of Ago1, Mov10 and GW182 in the same RNA samples using real time PCR. Ago1, which is a core protein in the RISC, did not show any changes after contextual conditioning. However, the expression of Mov10 increased from the early time point to 24 hours. Therefore, in addition to previous work, which showed that degradation of Mov10 protein is activity dependent [16], Mov10 transcription is also activity dependent. Transcript levels of GW182 decreased at 24 hours after contextual conditioning, but increased with NMDA activation and showed up- and then down-regulation with bicuculline treatment (Figure 6A and B). GW182 proteins repress translation, promote mRNA decay and induce P body formation to stabilize Ago proteins [42], all of which may contribute to regulatory controls at the 24 hour time point.

### 3′ UTRs of transcripts up-regulated after contextual conditioning have more predicted miRNA binding sites than down-regulated ones

**Figure 7:**
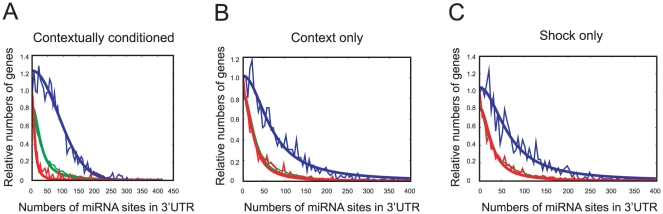
Up-regulates genes with more predicted miRNA sites. (A) Number of predicted miRNA binding sites in the 3′ UTR of genes up-regulated or down-regulated after fear conditioning (FC) (B) after context exposure only (C) shock only (microarray data from Peleg et al. (2010), Blue; up-, Red; down- regulated genes after FC, Green; every gene in Targetscan as a control.). (p<0.05, ANOVA, home cage: n = 12, 1 h and 3 h: n = 12, 24 h: n = 8. mean ± S.E.M.).

Peleg and colleagues reported microarray data of mRNAs in mouse hippocampus after contextual conditioning [43]. We looked for a potential miRNA regulation signature in that data. We assayed the number of predicted miRNA binding sites (Targetscan) in the 3′ UTR of those genes. 3′ UTRs of down-regulated transcripts in the context and shock controls have 28 and 31 predicted sites, respectively. These numbers of predicted miRNA binding sites do not differ significantly from the number of predicted miRNA sites in the 3′ UTRs from the entire mouse genome (33 binding sites). After contextual conditioning, the 3′ UTRs of up-regulated transcripts were twice as long as down-regulated transcripts [43]. 3′ UTRs of down-regulated transcripts have a mean length of 335 base pairs (bp) containing nine predicted miRNA binding sites, (3.6 fold fewer sites than in the 3′ UTRs of the entire mouse genome which have a mean length of 451 bp), whereas 3′ UTRs of up-regulated genes have a mean length of 1630 bp containing 83 predicted miRNA binding sites (Figure 7A to C), i.e. 2.5 fold more sites than in the 3′ UTRs of the entire mouse genome. This 9-fold difference in the number of miRNA binding sites together with difference in 3′ UTR length suggests that the down-regulated transcripts are less likely to be regulated by the miRNA machinery compared to up-regulated transcripts. This observation is consistent with reports that 3′UTR of neuronal genes are longer than ones in other types of cells [44].

### miR-19b changes mTOR activity in hippocampal neurons

**Figure 8:**
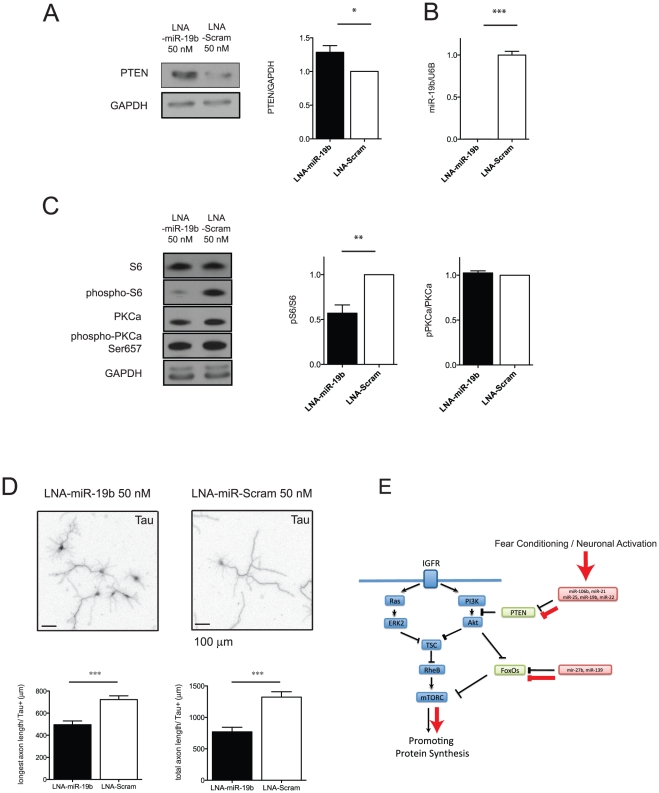
miR-19b regulates mTORC1 activity in neurons. Cultured hippocampal neurons were transiently transfected with either complementary sequence based locked nucleic acid inhibitors of miR-19b or a scrambled control in DIV1 (50 nM). In DIV5, RNA and protein lysates were collected and mTOR activity as well as the level of miR-19b was measured. (A) Western blot of PTEN as a miR-19b target gene. About 30% of protein is increased in miR-19b knock down neurons. *p<0.05, t-test, n = 7 from seven different biological samples. (B) Level of miR-19b was less than 0.1% in DIV5 neurons. n = 6 from three different biological samples, Student’s t-test, *** p<0.001. (C) Western blot of genes in the mTOR pathway. Inhibition of miR-19b reduced phospho-S6/S6 ratio representing mTORC1 activity. Inhibition of miR-19b didn’t change phospho-PKCa/PKCa ratio, representing mTORC2 activity. Student’s t-test ** p<0.01, n = 8 from 4 different biological samples. (D) Tau+ staining of neurons after 5 days of transfection. Longest and total neurite lengths were reduced in miR-19b knock down neurons. Scale bar  = 100 µm, Student’s t-test, *** p<0.001, n = 50 neurons per group. (E) Model: miRNAs that are up-regulated after contextual conditioning and neuronal stimulation regulate genes in the mTOR pathway. Red arrow indicates miRNA related mTOR pathways activated after contextual conditioning and neuronal stimulation.

To confirm that fear conditioning induced miRNAs that regulate mTOR signaling, we reduced the level of mature miR-19b expression in hippocampal neurons using a complementary sequence-based modified oligonucleotide inhibitor (locked nucleic acid, LNA) of miR-19b. We transiently transfected LNA inhibitors to DIV1 neurons and inhibition of miR-19b was measured using real time PCR. We confirmed that we could reduce expression of miR-19b to less than 0.1%, and inhibition lasted more than 4 days in culture (Figure 8B). Reducing miR-19b expression increased level of PTEN protein as reported previously [37] and down regulated phosphorylation of the S6 ribosomal subunit, which is thought to reflect mTORC1 activity (Figure 8A and C). In contrast, reducing miR-19b expression did not change the phosphorylation of PKCa, which reflects mTORC2 activity (Figure 8C). Finally, we showed that miR-19b suppression can phenocopy the morphological effects of inhibiting mTOR signaling by over-expressing TSC1 and 2, two proteins known to regulate of mTOR [45]. Rat hippocampal neurons at DIV5 have extended a set of neuritis; however, miR-19b was knocked down, early neurite outgrowth was significantly impaired (Figure 8D).

## Discussion

In the behavioral paradigm, contextual conditioning, we observed that about half of the profiled miRNAs underwent changes in their CA1 hippocampal expression from 1 h to 24 h after contextual conditioning. These changes were similar in amplitude to the response of *in vitro* cultured rat embryonic hippocampal neurons after chemical stimulation with NMDA or bicuculline. Interestingly, of 14 miRNAs that increased their expression after contextual conditioning and neuronal stimulation, seven of them have validated target genes important for mTOR activity. All seven of these miRNAs regulate inhibitors of the mTOR pathway. Therefore, their induction should lead to increases in mTOR signaling activity and, in fact, when one of them (miR-19b) was suppressed, its known target PTEN was increased and mTOR activity decreased. Suppression of miR-19b also reduced neurite outgrowth in DIV5 hippocampal neurons consistent with decreased mTOR activity. Accordingly, previous results showed that this signaling pathway is involved in learning and memory [46], [47]. Given the role of mTOR in the regulation of protein synthesis, this finding implies that one facet of the miRNA response to neuronal stimulation is to increase protein synthesis by de-repressing mTOR activity. Thus, de-repression of mTOR activity could serve as one explanation for the often noted paradoxical observation that upregulation of miRNAs can increase the levels of some proteins. mTOR plays a key role in shaping neuronal morphology and in memory processes [29], [48]. Our data suggest that miRNAs play a role in memory formation through mTOR activity and protein synthesis (Figure 8E).

Changes in miRNA expression correlate with contextual conditioning. At least two different stages are suggested by the kinetics of the expression changes: first, a group of miRNAs showed transient changes in expression peaking at 1–3 h after contextual conditioning. 73% of the miRNAs whose levels were significantly changed at 1 h were also changed at 3 h. Another group of miRNAs exhibited longer lasting expression changes; these were sustained until 24 h after training. 42% of the miRNAs that changed at 24 h showed the onset of change at either at 1 or 3 h.

The expression of miRNAs appears to be tightly regulated mechanism, perhaps to the level of single copy resolution at individual synapses. In our study, the expression of 11 precursor miRNAs (pri-miRNAs), was overall more constant throughout the learning paradigm than the mature miRNAs. This result suggests that the changes in miRNA expression are downstream of miRNA transcription in the biogenesis or degradation pathway. Indeed, transcript and protein levels of several core miRNA biogenesis components were affected by FC. Generation of the mature miRNA critically involves Dicer, Drosha and the Drosha partner, DGCR8 [49]; however, they did not change coordinately after FC (Figure 6). The differential changes in these transcripts may affect miRNA biogenesis due to changes in ratio between these two proteins as suggested by Shiekhattar et al [41]. Both proteins have been implicated spine development and cognitive performance [23], [25].

The fact that significant changes in miRNAs occur after learning, and that these changes are highly regulated suggests that changes in miRNA expression levels are involved in learning. This point is supported by the finding that up regulated genes after contextual conditioning have longer 3′ UTRs with 2.5 fold more predicted miRNAs binding sites than other genes whose expression is unchanged after learning. Conversely, downregulated genes had shorter 3′ UTRs with 3.6 fold fewer predicted miRNA binding sites. Thus, miRNAs appear to be regulating genes, which are implicated in memory formation more by releasing mRNAs from miRNA-mediated destabilization than by miRNA-mediated down-regulation of transcripts.

Further support for a key role in miRNA biogenesis in learning and memory comes from a knock down of the core RISC protein, Ago2, in a rodent model [24]. In a fascinating counter-intuitive result, the induction of *Dicer1* gene deletion in adult mouse forebrain increased learning ability for a period of time until neurodegeneration set in [23]. Very likely, the de-repression of local protein translation enhanced learning, released the latent capacity for spine growth in this study. In a similar vein, the release of local protein translation after activity-dependent stimulation was stimulated by Mov10 degradation [16]. Consistent with this result we show here that Mov10 transcript levels increased after contextual conditioning at 1 to 24 h, suggesting that activity dependent transcription replenishes the miRNA complex. In unpublished data, we found that Mov10 protein levels are restored at about 3 hours after depolarization (Bannerjee S and Kosik KS).

In summary, we have shown that expression levels of most miRNAs are altered in an NMDA-dependent manner in the hippocampal CA1 region of contextual conditioned mice and stimulated cultured embryonic rat hippocampal neurons. Those changes are probably due to altered levels of miRNA biogenesis pathways. Modifying the entire miRNA expression landscape is probably necessary to accommodate the thousands of genes upregulated after contextual conditioning. At synaptic sites the synthesis of locally made proteins are, in part, translationally repressed by miRNAs. Interestingly, the levels of these regulatory miRNAs themselves are controlled post-transcriptionally. The spatial and temporal regulation of gene expression in neurons is partly due to miRNA mechanisms, which also regulate memory formation.

## Materials and Methods

### Subject and contextual conditioning

Three months old male F1 hybrid mice (C57Bl/6NTac ×129S6/SvEvTac; Taconic Farms, Inc.) mice were used in this study. Initially, mice were group housed with free access to food and water and maintained on a 12∶12 h light:dark cycle in the Herbert L. Washington Vivarium in the Department of Psychology at UCLA. (UCLA approved animal protocol number 1999-107) A week before contextual conditioning training, mice were single housed and each mouse was handled for two minutes every day. Mice were also habituated to the holding room for contextual conditioning every day to exclude any factors other than contextual conditioning that could affect miRNA profiles. All experiments were performed during the light phase of the cycle.

During contextual conditioning, the mice were allowed to explore the training chamber for 2 minutes before the first shock was delivered. Mice received three footshocks (2 sec, 0.75 mA) with a one minute interval between each shock. One minute after the last shock, the mice were returned to their home cage. Contextual fear memory was assessed at 1 h, 3 h, or 24 h after training by placing the mice in the same training chamber for 3 minutes. Freezing behavior (defined as complete lack of movement, except for respiration) was scored automatically with the software (Med Associates). For some experiments, NMDA receptor (NMDAR) antagonist (±)-3-(2-Carboxypiperazin-4-yl) propyl-1-phosphonic acid (CPP, 20 mg/kg, i.p.; Sigma) or vehicle (saline) was administered to mice 30 min before the training. Dorsal CA1 was dissected as previously described [50] and immediately snap frozen at different time points as indicated in the results.

All procedures were approved by the Chancellor's Animal Research Committee at the University of California at Los Angeles, in accordance with US National Institutes of Health guidelines.

### RNA extraction

Total RNA was extracted using miRVana total RNA isolation kit (Ambion) according to manufacturer's instruction. RNA amount was measured using Nanodrop (Thermo Scientific).

### cDNA synthesis and real time PCR for miRNA

187-plexed real time PCR was used to measure expression of individual miRNAs. 40 ng of total RNA was reverse transcribed using cDNA Archiving Kit (Applied Biosystems) and 2.5 nM miRNA specific reverse primers. cDNA was pre-amplified using a common reverse primer (UR) and miRNA specific forward primers using Universal Master Mix with no UNG (Applied Biosystems). PCR product was diluted 4 times and 0.1 microliters was used for real-time PCR. Real-time PCR was performed in 7500HT fast real time System (ABI). To measure individual miRNA expression, we used Taqman® microRNA assays (ABI). Data was collected and analyzed as previously described [17].

### Hippocampal Cell Culture

All experimental procedures were performed in compliance with animal protocols approved by the IACUC at Children's Hospital, Boston. (Animal protocol number: A3303-01) Hippocampi were dissected from E18 Sprague-Dawley rat embryos (Charles River). Neurons were dissociated with Papain, triturated, and plated on poly-D-lysine/Laminin coated plates. Cells were plated at 250,000 cells/6 well plates for biochemistry and 20,000 cells/24 well plates for immuno staining experiments. Neurons were cultured in neurobasal medium with B27 supplement, 500 µM L-glutamine, 1x pen-strep at 37°C in a humidified incubator with 5% CO2.

### Western blot analysis for miRNA biogenesis genes

Immunoblot was performed from cell lysates of stimulated DIV15 neurons. Antibodies were used against DGCR8 (ab82876, 1∶500 dilution, Abcam), Drosha (ab58589, 1∶500 dilution, Abcam) and GAPDH (AM4300, 1∶5000 dilution, Ambion).

### miRNA inhibition in cultured neurons

We designed inhibitors of miRNA using Locked Nucleic Acid technology. (LNA, Exiqon) LNA sequences to inhibit expression of miR-19b is +T +C +A +G +T TTTGCATGGATTT +G +C +A +C +A, for the negative control, we used +C +A +T +T +A ATGTCGGACAAC +T +C +A +A +T. (“+”  =  locked base) We transiently transfected LNA oligos using lipofectamine 2000 and Optimen (Invitrogen) in DIV1 neuron. We followed the manufacturer's instructions. We measured inhibition of miR-19b using real time PCR.

### Measuring mTOR activity and neuronal morphology

Four days after transfection, we collected protein lysate and measured mTOR activity using western blots. PTEN (#9559, 1∶500 dilution, Cell Signaling), Phospho-S6 (#2211, 1∶1000 dilution, Cell Signaling), S6 (#2317, 1∶1000 dilution, Cell Signaling), phospho-PKCa (06-822, 1∶1000 dilution, Millipore), PKCa (#2056, 1∶1000 dilution, Cell Signaling) antibodies were used to measure miR-19b target and mTOR activity. GAPDH was used as a loading control. (AM4300, 1∶5000 dilution, Ambion) Neurons were fixed and stained with Tau antibody (MAB3420, 1∶800 dilution, Millipore) to measure axonal morphology. Tau positive neurites were measured using ImageJ.

### cDNA synthesis and real time PCR for pri-miRNAs and mRNAs

120 ng of total RNA was reversed transcribed using cDNA Archiving Kit with random primers (Applied Biosystems). For pri-miRNAs primer positions are usually ∼500 bp upstream from the mature miRNA and, according to the manufacturer, are unique genomic sequences. Power SyBr Green PCR Master Mix (Applied Biosystems) was used to amplify signals with 10 ng of cDNA and 1 µM of each primer. For pri-miRNA detection, we used TaqMan®Pri-miR Assays (Applied Biosystems). Sequences of primers for mRNA detection are; GAPDH-F 5′-aactttggcattgtggaagg-3′, GAPDH-R 5′-acacattgggggtaggaaca-3′, Arc-F 5′-agcagcagacctgacatcct-3′, Arc-R 5′-gtgatgccctttccagacat-3′, DGCR8-F 5′-gctgcaggagtaaggacagg-3′, DGCR8-R 5′-tcgagcactgcatactccac-3′, Drosha-F 5′-ggaccatcacgaaggacact-3′, Drosha-R 5′-gatgtacagcgctgcgataa-3′, mDicer-F 5′-gtggagggagaccagtcaaa-3′, mDicer-R 5′-tgggaagctatgggttcttg-3′, Ago1-F 5′-tcggaagatttccaaggatg-3′, Ago1-R 5′-gttgccattcccaagagtgt-3′, mMov10-F 5′-ttacccacccatcctcctc-3′, mMov10-R 5′-aggtcatagtgccggatgtc-3′, GW182-F 5′-agccttctactccagccaca-3′, GW182-R 5′-ggcccagatttgcttaatga-3′, rDicer-F 5′-gtacacctgccagaccacct-3′, rDicer-R 5′-tgaggtggttcagggttttc-3′, rMov10-F 5′-gcatggactcggctactctc-3′ rMov10-R 5′- aggcctgtagctctccatca-3′ (r; for rat neuron, m; mouse neuron, otherwise, we used same primers). Amplified signals were collected by 7500HT fast real time System (ABI) and normalized with GAPDH.

### Data analysis

All data analysis was done using custom-written scripts in Python using SciPy and NumPy modules.

### Normalization

Ct values for each sample are globally normalized using all Ct values below 25. miRNAs which are detected with a Ct value <30 in at least one sample were kept for further analysis. To correct for variations due to different batches of reagents, 4 control animals were always profiled with each new reagent batch and Ct values were corrected for individual miRNAs using the mean miRNA abundance computed over the 4 animals for the different reagent batches.

### Statistical analysis

Distributions of coefficient of variation were compared using the Kolmogorov-Smirnov test. miRNAs that changed significantly between two conditions were determined using the Kruskal-Wallis test. Significance level was set at 0.05.

### 3′UTR analysis

We used the mouse UTR database of Targetscan. The distribution of UTR lengths was well approximated by a Lorentz distribution. For a given set of UTRs, we use the half width at half maximum as a measure of the characteristic length associated with that set.

## Supporting Information

Figure S1 (A) miRNAs changing at early time points (1 and 3 h after FC). (B) miRNAs showing expression changes at later time points (3 and 24 h after FC). (C) miRNAs with long-lived expression changes (1 to 24 h after FC).
(EPS)

Figure S2 miRNAs with expression significantly different from the one in unstimulated neurons. (p<0.05, Kruskal-Wallis test) 32 miRNAs showed expression changes in common after NMDA and bicuculline stimulation. (unstimulated; n = 10, NMDA; n = 10, 60 μM, 5 min, bicuculline; n = 8, 20 μM, 1 hour)
(EPS)

Data S1 miRNA profiling after contextual conditioning in mouse hippocampal CA1 region
(XLSX)

Data S2 miRNA profiling after neuronal activation in embryonic cultured neuron
(XLS)
